# GLI1-mediated regulation of side population is responsible for drug resistance in gastric cancer

**DOI:** 10.18632/oncotarget.16174

**Published:** 2017-03-14

**Authors:** Beiqin Yu, Dongsheng Gu, Xiaoli Zhang, Jianfang Li, Bingya Liu, Jingwu Xie

**Affiliations:** ^1^ Shanghai Key Laboratory of Gastric Neoplasms, Shanghai Institute of Digestive Surgery, Ruijin Hospital, Shanghai Jiao Tong University School of Medicine, Shanghai 200025, China; ^2^ Department of Pediatrics, The Wells Center for Pediatrics Research and IU Simon Cancer Center, Indiana University School of Medicine, Indianapolis, IN 46202, USA

**Keywords:** GLI1, hedgehog, cisplatin, gastric cancer, chemoresistance

## Abstract

Gastric cancer is the third leading cause of cancer-related mortality worldwide. Chemotherapy is frequently used for gastric cancer treatment. Most patients with advanced gastric cancer eventually succumb to the disease despite some patients responded initially to chemotherapy. Thus, identifying molecular mechanisms responsible for cancer relapse following chemotherapy will help design new ways to treat gastric cancer. In this study, we revealed that the residual cancer cells following treatment with chemotherapeutic reagent cisplatin have elevated expression of hedgehog target genes *GLI1*, *GLI2* and *PTCH1*, suggestive of hedgehog signaling activation. We showed that *GLI1* knockdown sensitized gastric cancer cells to CDDP whereas ectopic *GLI1* expression decreased the sensitivity. Further analyses indicate elevated GLI1 expression is associated with an increase in tumor sphere formation, side population and cell surface markers for putative cancer stem cells. We have evidence to support that GLI1 is critical for maintenance of putative cancer stem cells through direct regulation of ABCG2. In fact, GLI1 protein was shown to be associated with the promoter fragment of *ABCG2* through a Gli-binding consensus site in gastric cancer cells. Disruption of ABCG2 function, through ectopic expression of an ABCG2 dominant negative construct or a specific ABCG2 inhibitor, increased drug sensitivity of cancer cells both in culture and in mice. The relevance of our studies to gastric cancer patient care is reflected by our discovery that high ABCG2 expression was associated with poor survival in the gastric cancer patients who underwent chemotherapy. Taken together, we have identified a molecular mechanism by which gastric cancer cells gain chemotherapy resistance.

## INTRODUCTION

Although gastric cancer is the third leading cause of cancer-related death worldwide [1–4], our basic understanding of gastric cancer falls behind that of many other cancer types. Clinically, gastric cancer is treated by surgical resection with chemotherapeutic interventions as major options [5]. Even with an increased enrollment rate for chemotherapy of gastric cancer, the overall median survival remains between 15 to 17 months [6]. Patients initially respond to chemotherapy but cancer eventually relapses. Therefore, chemotherapy resistance becomes a major barrier to achieve effective gastric cancer treatment. Thus, finding novel strategies to sensitize cancer cells to chemotherapy will significantly improve gastric cancer patient survival.

Chemotherapy in gastric cancer is generally used as multimodality treatment, such as perioperative and adjuvant chemotherapy [7, 8]. The common chemotherapeutical agents for gastric cancer include cisplatin (CDDP), 5-fluorouracil (5-FU) or its oral administered derivatives capecitabine and S-1. Common combinations include ECF (epirubixin, cisplatin and 5-FU), 5-FU plus either cisplatin or docetaxel (or irinotecan) with radiation [9, 10]. The mechanisms underlying chemoresistance in gastric cancer are not entirely known, but the following mechanisms have been reported: decreased intracellular drug accumulation and/or increased drug efflux, increased nucleotide excision-repair activity, evasion of apoptosis, activation of several signaling pathways and the existence of putative cancer stem cells. Another theory for chemosensitivity is the cancer stem cell hypothesis: a small percentage of cancer cells, the residual cancer cells or the putative cancer stem cells, are resistant to chemotherapy-mediated cell killing, and become the source for cancer relapse. If the regulatory mechanisms for maintaining this cell population are discovered, agents disrupting the mechanisms may be used to develop novel strategies to treat gastric cancer.

There are a number of signaling pathways involved in regulation of drug resistance, including the hedgehog pathway [11–15]. Hedgehog signaling is an important pathway for embryonic development, tissue patterning, cell differentiation, cancer development and drug resistance [16–18]. In the last few years, hedgehog signaling is reported in regulation of drug resistance in several types of cancer including ovarian, prostate and pancreatic cancer [11, 15, 19–21]. In hedgehog signaling active cells, the hedgehog ligands (Shh, Ihh or Dhh) bind to PTCH1, allowing smoothened to signal to the downstream GLI transcription factors, which then turn on the target genes [18]. Target genes of hedgehog signaling include GLI1, PTCH1 as well as other molecules involved in regulation of cancer cell function, such as ABCG2 [22]. ABCG2 is an important transportor and functions in maintenance of the stem cell population through side population regulation.

In this study, we report a novel mechanism underlying drug resistance in gastric cancer. We found that treatment of gastric cancer cells with the chemotherapeutical drug CDDP often results in elevated hedgehog (Hh) signaling which is associated with an increase in putative cancer stem cell markers. We further investigated the functional significance of Hh signaling and ABCG2 in cultured cells and in mouse models. The relevance of our findings to gastric cancer patients was further examined in human specimens. These findings may lead to novel strategies to improve the effectiveness of chemotherapy in gastric cancer.

## RESULTS

### Expression of Hh target genes following chemotherapeutical drug treatment

**Figure 1:**
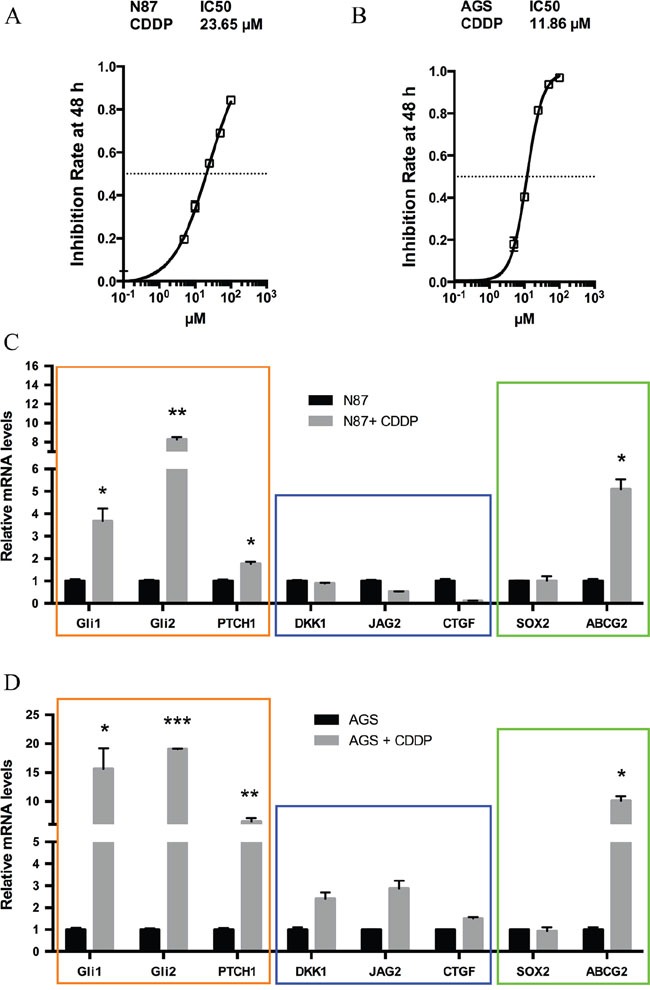
Elevated hedgehog signaling following CDDP treatment in gastric cancer cells **(A)** The inhibition rate of cell viability by different concentrations of CDDP in N87 cells were determined by chemosensitivity assay (see Methods), and the IC50 was calculated from this experiment. Cells were treated with increasing concentrations of CDDP (5, 10, 25, 50, 100 μM) for 48 hr. **(B)** The rate of cell viability inhibition by CDDP in AGS cells were also determined by chemosensitivity assay (see Methods), and we calculated the IC50 from this experiment. **(C)** The transcript levels of *GLI1*, *GLI2*, *PTCH1*, *DKK1*, *JAG2*, *CTGF*, *SOX2* and *ABCG*2 in N87 cells after treatment with 25 μM CDDP for 48 hr. **(D)** The transcript levels of above genes in AGS cells after 10 μM CDDP for 48 hr. Data are represents as mean ± SD from three independent experiments. * *P* < 0.05, ** *P* < 0.01, *** *P* < 0.001.

To delineate the molecular mechanisms underlying chemotherapy resistance in gastric cancer, we examined molecular changes following treatment with CDDP, the most common chemotherapeutical agent [23–25]. Two cancer cell lines N87 and AGS were used in our initial studies. We predicted that the residual cells with intrinsic drug resistance to chemotherapy will survive following drug treatment, and the responsible genes will be up-regulated. As a first step, we measured the IC50 of CDDP in gastric cell lines from the inhibition rate of cell viability by different concentrations of CDDP (Figure 1A and 1B). We then treated cells with CDDP at the IC50 dose for 48 hr and examined the candidate gene expression. Previous studies indicate many candidate genes involved in regulation of residual cancer cells or putative cancer stem cells, such as Hh, Wnt and Notch signaling molecules [26–28]. As shown in Figure 1C, we found that target genes for the Hh signaling pathway, *GLI1*, *GLI2* and *PTCH1*, were significantly induced in CDDP-treated N87 cells. Similar results were also obtained from AGS cells (Figure 1D). In contrast, we did not observe significant gene expression changes in *DKK1*, *JAG2* or *CTGF*, molecules involved in Wnt, Notch and Hippo/YAP signaling (Figure 1C and 1D, Supplementary Figure 1).

Like Wnt and Notch signaling, Hh signaling plays an important role in embryonic development, and is also critical for maintenance of putative cancer stem cells or residual cancer cells [26, 29, 30]. We thus examined expression of several putative cancer stem cell markers [31–38] following CDDP treatment in N87 cells. There are a number of factors involved in regulation of putative cancer stem cells [39–43]. For example, the side population is often enriched in stem cells and cancer stem cells, and ABCG2 is the major gene regulating side population [44]. Sox2 is another important factor involved in regulation of putative cancer stem cells [45]. Through real-time PCR analysis, we found high expression of *ABCG2* following drug treatment (Figure 1C). This phenomenon did not appear to be cell line specific because similar results were also observed in AGS cells (Figure 1D). In contrast, *SOX2* expression was not significantly changed (Figure 1C and 1D).

These results indicate that elevated Hh signaling may be responsible for maintenance of residual cancer cells (or putative cancer stem cells or tumor initiating cells) following chemotherapeutic drug treatment in gastric cancer.

### Significance of GLI1 expression for intrinsic drug resistance in gastric cancer cells

**Figure 2:**
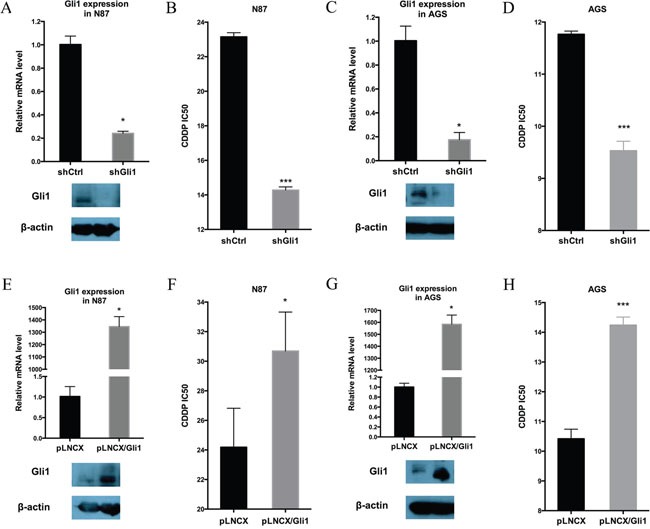
GLI1 expression is required and sufficient for intrinsic drug resistance in gastric cancer cells **(A)** GLI1 transcript level and the protein level in N87/shCtrl and N87/shGLI1 cells as determined by real-time PCR and Western blot analysis, respectively. **(B)** IC50 dose of CDDP in N87/shCtrl and N87/shGLI1 cells determined by chemosensitivity assay. **(C)** GLI1 transcript and protein levels in AGS/shCtrl and AGS/shGLI1 cells. **(D)** The CDDP IC50 dose in AGS/shGLI1 cells compared with AGS/shCtrl cells. **(E, F)** The effect of ectopic Gli1 expression on the IC50 of CDDP in N87 cell. **(E)** shows GLI1 transcript (upper) and protein (low) levels in N87 with or without ectopic GLI1 expression (pLNCX indicates the vector control, and pLNCX-Gli1 indicates ectopic Gli1 expression). **(F)** shows the IC50 values from pLNCX and pLNCX-Gli1 N87 cells. **(G, H)** The effect of ectopic Gli1 expression on the IC50 value of CDDP in AGS cells. **(G)** shows GLI1 transcript (upper) and protein (low) levels in AGS cells with pLNCX as the vector control and pLNCX/GLI1 as the ectopic Gli1 expression. **(H)** shows the IC50 values from pLNCX and pLNCX/GLI1. Means ± SD from three independent experiments are shown. * *P* < 0.05, ** *P* < 0.01, *** *P* < 0.001.

To evaluate the functional relevance of Hh signaling for the intrinsic drug resistance in N87 and AGS cells, we first knocked down *GLI1* expression by expressing *GLI1* shRNAs in both cell lines, and then determined the IC50 for CDDP. We found that down-regulation of *GLI1* in N87 cells (Figure 2A) reduced the IC50 by nearly half (Figure 2B). The IC50 value was also reduced by GLI1 knockdown in AGS cells (Figure 2C and 2D). Additional experiments in IC50 measurement and tumor sphere formation indicate that knocking down both GLI1 and GLI2 has similar effect as GLI1 knockdown (Supplementary Figure 2 for IC50 value, and Supplementary Figure 3 for tumor sphere formation), suggesting that the feed-forward loop exerted by GLI1 is the major factor for regulation of putative cancer stem cells. Thus, GLI1, the focus for the rest of our study, appears to be critical for drug resistance in gastric cancer cells.

Furthermore, we determined whether elevated Hh signaling is sufficient to drive drug resistance in gastric cancer cells by ectopic expression of *GLI1* in N87 and AGS cells, and examining their IC50 values for CDDP. We discovered that ectopic *GLI1* expression in both N87 and AGS cells significantly increased the IC50 value (Figure 2E-2H).

Taken together, we found that the *GLI1* expression level is highly associated with chemosensitivity in gastric cancer cells. While down-regulation of *GLI1* decreases the IC50, ectopic expression of *GLI1* increases the CDDP IC50.

### Regulation of the putative cancer stem cell population by GLI1

Previous studies have revealed heterogeneous cell populations even within the established cell lines [31–38], and our experiments with CDDP treatment studies also suggest that a subset of cells (e.g. putative cancer stem cells) are more resistant to drug treatment, and that GLI1 may play an important role in maintenance of this cell population. To directly test the role of GLI1 for putative cancer stem cell maintenance, we detected the putative cancer stem cell population in gastric cancer cells using three methods.

**Figure 3:**
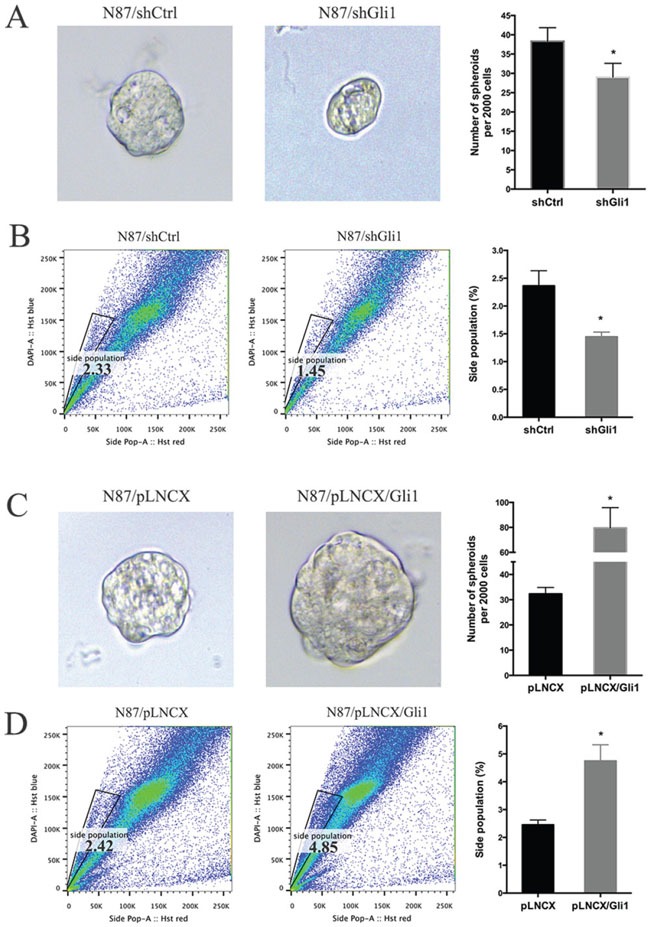
GLI1 regulates the putative cancer stem cell population in N87 cells **(A)** Representative images and statistical graph of N87/shCtrl and N87/shGLI1 cells grown as tumor spheres. **(B)** Side population in N87/shCtrl and N87/shGLI1 cells was measured by flow cytometry. Representative flow cytometric analysis was shown. **(C)** Representative images of spheres from N87/pLNCX and N87/pLNCX/GLI1 cells. **(D)** The percentage of side population in N87/pLNCX/GLI1 cell and the control cells. All data are means ± SD of three independent experiments. * *P* < 0.05, ** *P* < 0.01, *** *P* < 0.001.

First, we measured tumor sphere forming efficiency from N87 cells with *GLI1* shRNAs, ectopic *GLI1* expression or the control cells. Tumor sphere formation efficiency is a known biological readout of cancer stem cells [46]. We found that *GLI1* knockdown significantly reduced the size of tumor spheres (Figure 3A). *GLI1* shRNA expression also reduced the tumor sphere forming efficiency (Figure 3A, with the control ∼38 spheres /2000 cells and Gli1 knockdown cells 29 spheres/ 2000 cells, *P* = 0.032). Conversely, ectopic expression of *GLI1* increased the tumor sphere forming efficiency (Figure 3C, with the control ∼32.5 spheres/2000 cells and GLI1 expressing cells 80 spheres/ 2000 cells, *P* = 0.034). It thus appears that high GLI1 expression increases the tumor sphere forming efficiency.

Second, we also detected side population of cancer cells after GLI1 alteration. Side population is a functional assay for the transporter ABCG2, and is a well-known readout for stem cells and putative cancer stem cells [47–49]. We found that *GLI1* shRNA reduced the side population by 40% (Figure 3B) whereas ectopic *GLI1* expression increased the side population by 100% (Figure 3D).

Third, we also detected cell surface marker expression following alteration of GLI1 levels. Since there are no consensus cell surface markers for putative cancer stem cells in gastric cancer, we examined changes in the following markers: CD24, CD33, CD44, CD90 and CD133 that are reported in the literature [31–38]. We did not see significant changes in CD24, CD44 and CD33 in our experiments. However, we found that *GLI1* knockdown significantly reduced expression of CD90 and CD133 (Supplementary Figure 4A/B) whereas ectopic *GLI1* expression increased expression of CD90 (Supplementary Figure 4C/D). Although CD44 was reduced by GLI1 shRNAs and was reportedly regulated by hedgehog signaling [50], the decrease in our experiments did not reach statistical significance.

Taken all the data together, we concluded that GLI1 expression is an important factor for maintenance of the putative cancer stem cell population as indicated by tumor sphere forming efficiency and side population. While *GLI1* knocking down reduces the putative cancer stem cell population, ectopic expression of *GLI1* increases this population.

### The role of ABCG2 in drug sensitivity regulation in gastric cancer cells

**Figure 4:**
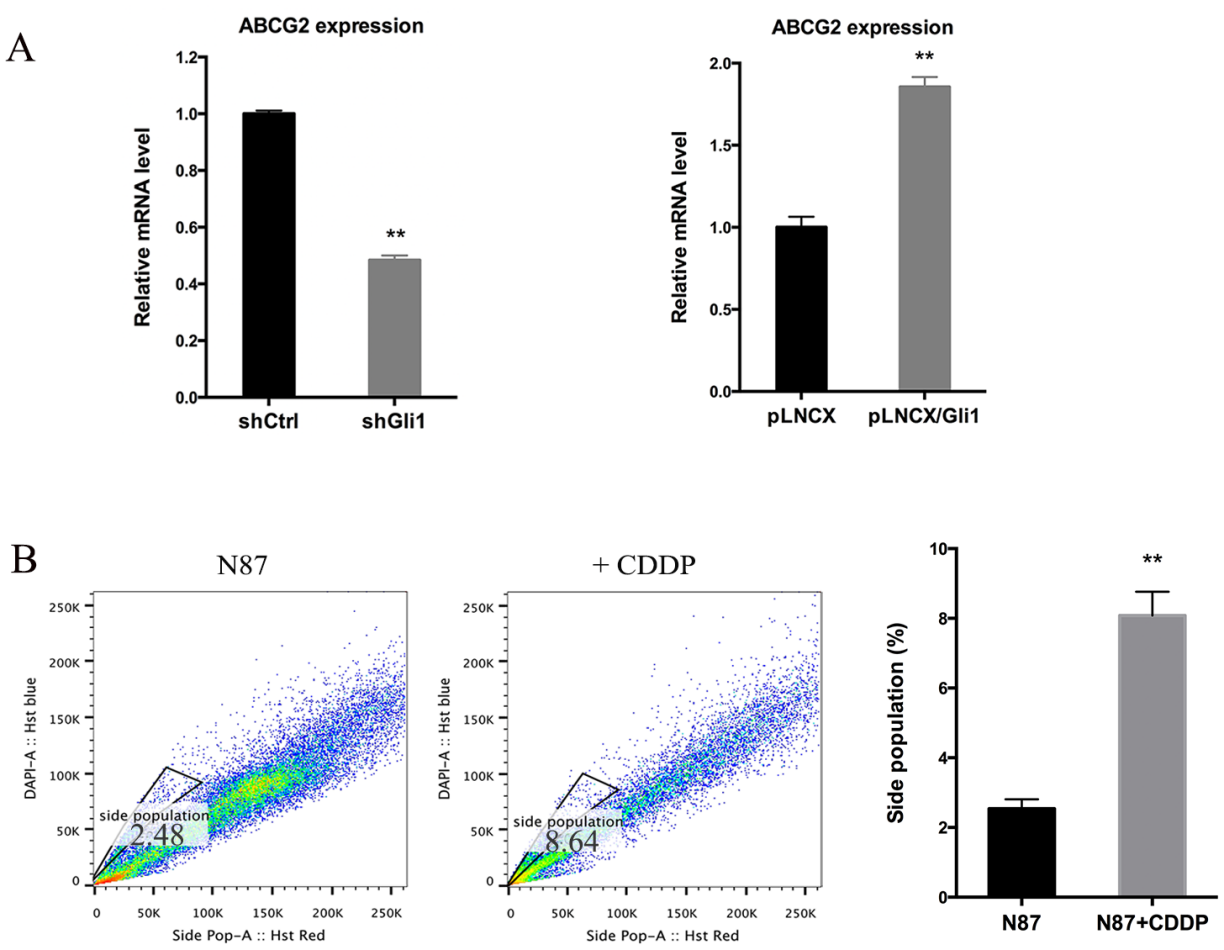
Regulation of *ABCG2* by GLI1 **(A)** The *ABCG2* transcript level in N87/shCtrl and N87/shGLI1 cells (left panel), N87/pLNCX and N87/pLNCX/GLI1 cells (right panel) as detected by real-time PCR. **(B)** Representative side population analysis and statistical graph of side population in N87 cells treated with 25 μM CDDP in comparison with control cells. Means ± SD are shown. ** *P* < 0.01.

Because ABCG2 is responsible for exclusion of Hoechst 33342 dye and the subsequent side population, we tested whether GLI1 directly regulate ABCG2 expression. Through real-time PCR analysis, we found that while *GLI1* shRNAs significantly reduced *ABCG2* expression by half, ectopic *GLI1* expression induced *ABCG2* transcript by 100% (Figure 4A). Similarly, treatment with CDDP increased expression of *ABCG2* (Figure 1C and 1D) and the side population (Figure 4B).

After promoter analysis, we discovered a GLI1-binding consensus site [18] in the promoter of *ABCG2* (Supplementary Figure 5C), suggesting that *ABCG2* may be a transcriptional target of GLI1 molecules. We performed ChIP analysis [51] following ectopic expression of *GLI1* in N87 and AGS cells (Supplementary Figure 5A and 5B). Supplementary Figure 5C showed the 9 base pairs sequence of the potential GLI1 binding site within the *ABCG2* promoter. Our data indicate that ectopically expressed GLI1 protein (with MYC tag) can be pulled down with the *ABCG2* promoter region encompassing the GLI1 binding site (Supplementary Figure 5D and 5E) whereas the control IgG does not pull down the fragment. Similarly, MYC antibodies did not pull down the fragment in cells without ectopic GLI1 expression. These results suggest that GLI1 may directly regulate ABCG2 expression by transcriptional regulation, which is consistent with a previous report in lymphomas [52].

**Figure 5:**
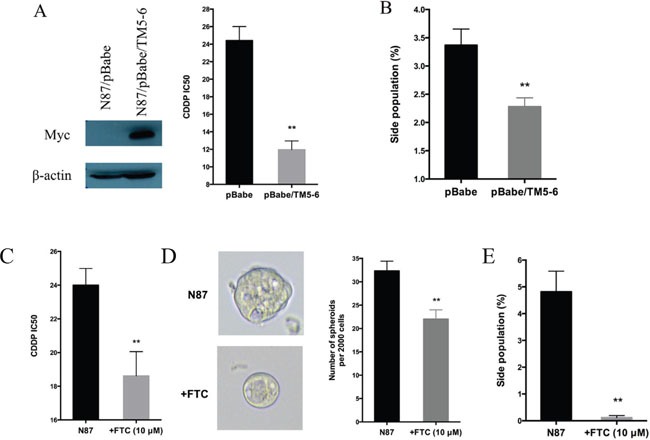
Regulation of drug sensitivity by ABCG2 **(A)** Effects of ABCG2-TM5-6, the truncated form of ABCG2 on drug sensitivity. ABCG2-TM5-6 was detected by the Myc-tag in Western blot analysis (left panel). The IC50 for CDDP in these two groups of cells were shown in the right panel. **(B)** Statistical graph of side population distribution in N87/pBabe and N87/pBabe/TM5-6 cells. **(C)** The IC50 of CDDP in N87 cells with or without FTC (10 μM). **(D)** Representative images and statistical graph of spheres in N87 cells treated or untreated with FTC. **(E)** Statistical graph of side population in N87 cells with or without FTC. The results are means of three independent experiments ± SD. * *P* < 0.05, ** *P* < 0.01.

**Figure 6:**
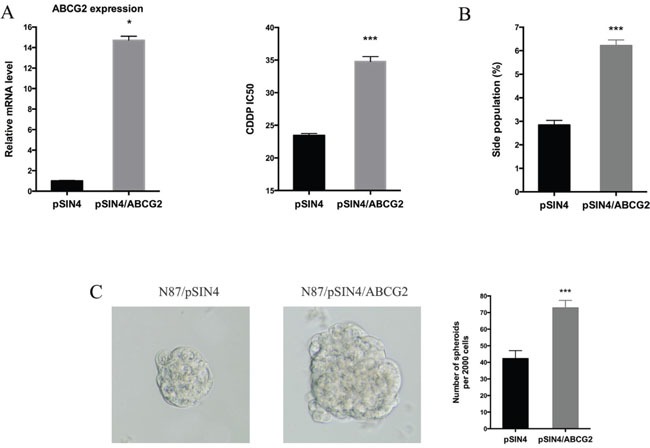
ABCG2 overexpression increases drug resistance **(A)** The *ABCG2* transcript level in N87/pSIN4 and N87/pSIN4/ABCG2 cells was detected by real-time PCR (left panel). The IC50 of CDDP was shown in the right panel. **(B)** Statistical graph of side population analysis in N87/pSIN4 and N87/pSIN4/ABCG2 cells. **(C)** Representative images of spheres (left two panels) and statistical graph of tumor spheres in N87/pSIN4 and N87/pSIN4/ABCG2 cells. Means ± SD are shown. * *P* < 0.05, *** *P* < 0.001.

Next, we evaluated the functional relevance of ABCG2 for regulating drug sensitivity. We used two methods to reduce ABCG2 function. First, we used a truncated form of ABCG2, which has a dominant negative effect on the endogenous *ABCG2* gene function [53], and found that ectopic expression of this truncated form of ABCG2 reduced the IC50 of CDDP by half (Figure 5A), which was associated with reduced side population (Figure 5B and Supplementary Figure 6A) and a decrease in CD90 expression (Supplementary Figure 6B). Similarly, when an ABCG2 inhibitor FTC [54] was used, we observed a significant reduction in the IC50 (Figure 5C), sphere formation (Figure 5D) and side population (Figure 5E, Supplementary Figure 6C). Conversely, we found that overexpression of *ABCG2* increased the IC50 for CDDP (Figure 6A), which was associated with an increase in side population (Figure 6B), sphere formation (Figure 6C and Supplementary Figure 6D) and CD90 expression (Supplementary Figure 6E).

The effect of ectopic ABCG2 expression on the IC50 of CDDP was very similar to that GLI1 expression, suggesting that ABCG2 may be the major mediator for regulating drug sensitivity in gastric cancer. In consistent with our hypothesis, ectopic expression of *ABCG2* had no significant effects on expression of *GLI1*, *GLI2* and *PTCH1* in both N87 and AGS cells (Supplementary Figure 7), further confirming that hedgehog signaling is upstream of ABCG2 in our experiment system.

### Therapeutic effects of CDDP in ectopic expression of GLI1 xenograft model

**Figure 7:**
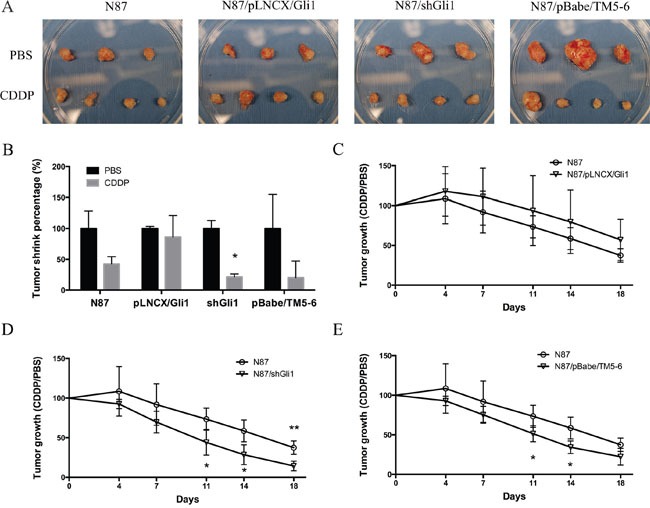
CDDP sensitivity in mouse models **(A)** Photographs of tumor xenografts derived from N87, N87/pLNCX/GLI1, N87/shGLI1 or N87/pBabe/TM5-6 in NSG mice treated with PBS (top) or CDDP treatment (bottom). **(B)** Tumor weights shrink percentage after CDDP treatment compared with PBS control in four different xenografts groups. **(C)** The growth curves of tumor xenografts in mice during CDDP treatment in N87 and N87/pLNCX/GLI1 groups. **(D)** The tumor growth in N87/shGLI1 group and the N87 control group during CDDP treatment. **(E)** Tumor growth curves in N87 and N87/pBabe/TM5-6 groups. Means ± SD are shown. * *P* < 0.05, ** *P* < 0.01.

To determine the relevance of our *in vitro* studies to therapeutic implication, we tested the effects of CDDP on the subcutaneous mouse model using N87 cells with alterations in *GLI1* and *ABCG2. GLI1* was ectopically expressed in N87/pLNCX/GLI1 cells while *GLI1* expression was knocked down in N87/shGLI1 cells. A truncated form of ABCG2 was expressed in N87/pBabe/TM5-6 cells. As shown in Figure 7A and 7B, CDDP treatment reduced the tumor significantly in the N87 control group. In contrast, ectopic *GLI1* expression in N87 cells did not respond to CDDP treatment whereas *GLI1* shRNA expression sensitized tumors to CDDP treatment. Blockage of ABCG2 function, via ectopic expression of a dominant negative construct ABCG2/TM5-6, made N87 gastric cancer cells more sensitive to CDDP treatment (Figure 7B). Tumors formed from the cells expressing shGLI1 or TM5-6 reduced the tumor size significantly following CDDP treatment (Figure 7D and 7E). In contrast, tumors formed from the cells with ectopic *GLI1* expression were not significantly affected by CDDP (in compared with the N87 control group, Figure 7C). These results are consistent with the *in vitro* studies, further confirming that the levels of GLI1 and ABCG2 expression determine the sensitivity of gastric cancer cells to CDDP treatment.

### Higher ABCG2 expression in gastric cancer patients with poor prognosis

**Figure 8:**
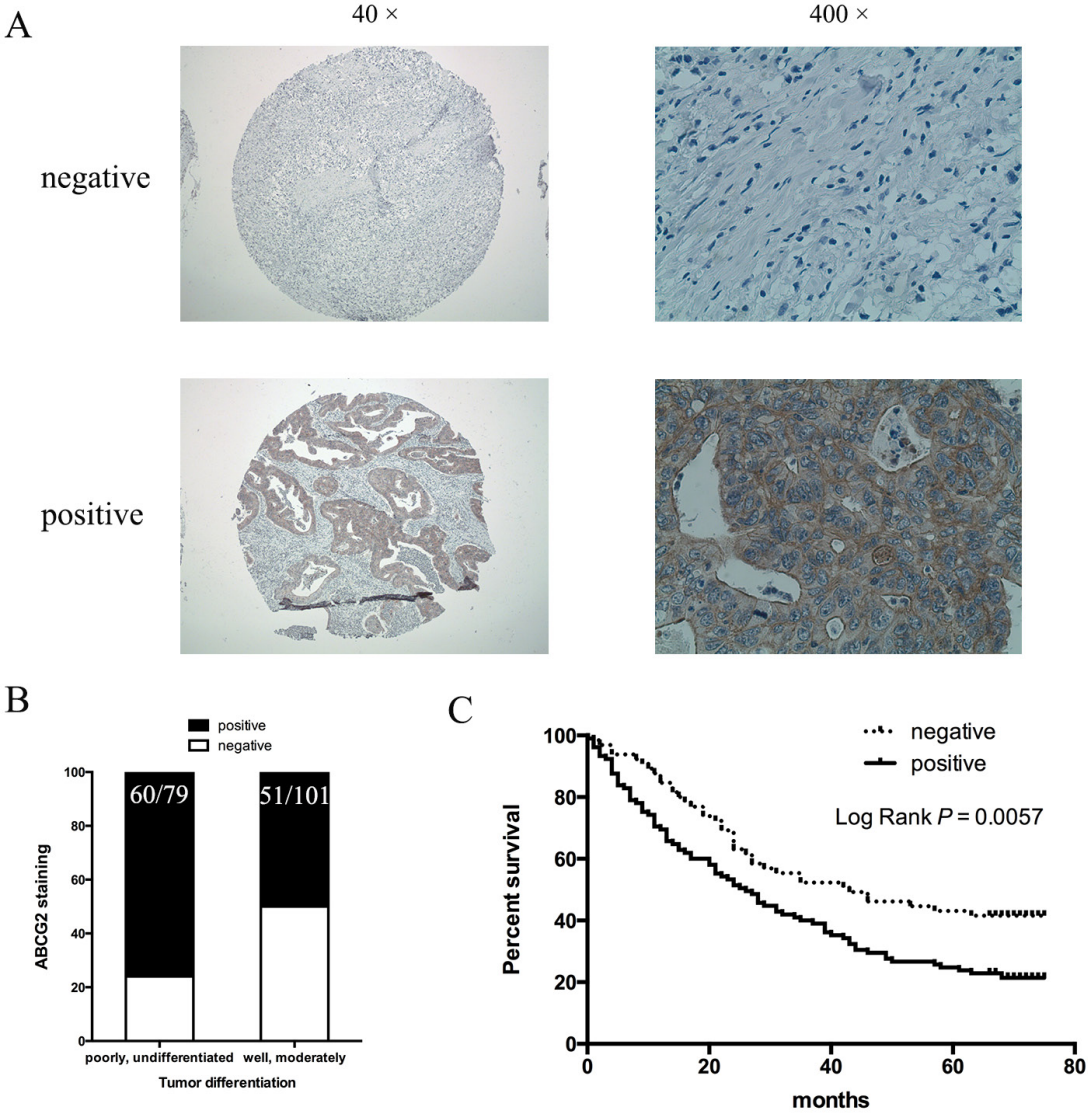
Correlation of ABCG2 expression in gastric cancer with tumor differentiation and patient survival **(A)** Representative images of immunohistochemical staining of ABCG2 in gastric cancer specimens. Original magnification: 40× and 400×. **(B)** Staining of ABCG2 and correlation with tumor differentiation (*P* = 0.0005). **(C)** Kaplan-Meier analysis of the significance of ABCG2 in predicting gastric cancer patient overall survival. *P* = 0.0057 by log rank test.

We went further to find the relevance of our data to gastric cancer patients by correlating ABCG2 expression in the tumor with the survival of patients who underwent chemotherapy using CDDP. The expression level of ABCG2 was analyzed by immunohistochemistry in 180 cases of gastric cancer patients who underwent chemotherapy. According to our criteria (see Materials and Methods), 62% (111/180) of gastric cancer specimens were positively stained with anti-ABCG2 antibodies (Figure 8A). We found that positivity of ABCG2 staining was significantly correlated with clinicopathological parameters. As shown in Table 1, the presence of ABCG2 was higher in poorly differentiated gastric cancer specimens in comparison with well-differentiated ones (Figure 8B, *P* = 0.0005). There was no difference in the correlation between ABCG2 expression and gender, age, tumor size and tumor location (see Table 1 for details). Among these 180 gastric cancer patients, 170 cases were available with the follow-up information. As Figure 8C shown, higher ABCG2 expression in gastric cancer was correlated with a shorter overall survival, indicating that high ABCG2 expression in the tumor predicts a poor outcome in chemotherapy in gastric cancer patients.

**Table 1:** Relationship between ABCG2 membrane staining and clinicopathologic features in 180 gastric cancer tissues

Clinicopathologic parameters	ABCG2 expression		*P*-value
	negative (n = 69)	positive (n = 111)	
Gender			
Male	46	77	0.7047
Female	23	34	
Age (years)			
≤ 60	35	41	0.0686
>60	34	70	
Tumor size (cm)			
≤ 5	36	55	0.7321
>5	33	56	
Location			
Distal third	41	59	0.4107
Middle third, proximal third	28	52	
Differentiation			
Poorly, undifferentiated	19	60	0.0005
Well, moderatelly	50	51	
Local invasion			
T1, T2	9	16	0.7960
T3, T4	60	95	
Lymph node metastasis			
No	19	27	0.6310
Yes	50	84	
TNM stage			
I, II	28	41	0.6250
III, IV	41	70	

Because there are no GLI1 antibodies suitable for immunohistochemistry in paraffin-embedded tissues, we took the advantage of the CTGA data to test whether higher GLI1 transcript level is correlated with cancer relapse or patient survival in those patients who underwent chemotherapy. In a cohort of 415 cancer specimens, we found that 7 out of 12 (>50%) patients with high GLI1 transcript had relapsed cancer whereas 102 out of 310 patients with low GLI1 expression had relapsed cancer. In addition, patients with high Gli1 expression had worse disease-free survival (Supplementary Figure 9). These results suggest that the GLI1-ABCG2 signaling axis is associated with poor outcomes of the gastric cancer patients.

From all these data, we conclude that activation of Hh signaling, through regulation of ABCG2, plays an important role in regulation of drug sensitivity in gastric cancer. We predict that the novel strategies aimed at reducing GLI1 expression or interrupting ABCG2 function, together with CDDP-based chemotherapy, should improve the survival of gastric cancer patients.

## DISCUSSION

Gastric cancer remains a major contributor for cancer-related mortality. Most patients are diagnosed with advanced disease where the five-year survival rate is very low (< 5%) [1]. Although the regulatory mechanisms for chemotherapy resistance in gastric cancer have been reported in the last 10 years [55], very little data have been linked the mechanisms to patient survival. Our results indicate that the GLI1-mediated regulation of ABCG2 is an important mechanism responsible for maintenance of the residual cancer cells (or putative cancer stem cells) in gastric cancer following treatment with CDDP, a major chemotherapeutical drug. We have data to show that knocking down *GLI1* (Figure 2 and Figure 3) or inhibiting ABCG2 functions (Figure 5), sensitizes cancer cells to chemotherapy in cultured cells (Figure 2-6) and in mice (Figure 7). More importantly, we have shown that higher GLI1/ ABCG2 expression is associated with poor survival of gastric cancer patients who underwent CDDP-based chemotherapy (Figure 8 and Supplementary Figure 9). Since inhibitors for GLI1 and ABCG2 are already available, we predict that these agents, together with CDDP-based chemotherapy, will improve gastric cancer patient survival.

Like Wnt and Notch signaling pathways, Hh signaling is known to be a critical regulator for embryonic development, and is involved in maintenance of the cancer cell stemness in a number of cancer types [18, 26, 29, 30, 56]. There are several reports on association of Hh signaling with drug resistance [13, 56, 57], but the molecular link is often not known. Yoon *et al* reported a link between Hh signaling and CD44 expression in a number of cell lines through sphere formation studies, which was shown to be associated with cisplatin resistance in gastric cancer [50]. While we have confirmed regulation of CD44 by *GLI1* expression, we found that GLI1 affects ABCG2 more dramatically, with little effects on CD44 (Supplementary Figure 4). Furthermore, we demonstrated that inhibition of ABCG2 by expressing a truncated ABCG2 molecule or addition of a specific inhibitor, sensitizes cancer cells to CDDP treatment (Figure 5). We found that the ligands, Shh and Ihh, were not significantly altered by CDDP in N87 and AGS cells (Supplementary Figure 8), suggesting that up-regulation of *GLI1* in these cells was not caused by ligand-dependent signaling. This implies that the SMO inhibitors, with two already approved by FDA for basal cell carcinoma treatment [58], will not be effective in sensitizing gastric cancer cells to chemotherapy. It will be interesting, though, to reveal the molecule mechanisms by which *GLI1* expression is induced in cancer cells with intrinsic tolerance of CDDP, which may provide additional strategies to sensitize gastric cancer cells to chemotherapy. Targeting ABCG2 will be a more feasible strategy to improve the sensitivity of gastric cancer cells to chemotherapy. Although ABCG2 is known to be responsible for transporting several types of small molecules, such as ions, dyes and some chemotherapeutical drugs, CDDP transport in and out of cells is not affected by ABCG2 [59, 60], further confirming that ABCG2 expression may represent a subset of cell population (side population) with cancer cell stemness.

## MATERIALS AND METHODS

### Cell culture

Human gastric cancer cell lines AGS and N87, and HEK293T were purchased from ATCC. Gastric cancer cells were maintained in RPMI-1640, while HEK293T cells were cultured in DMEM, supplemented with 10% FBS, 100 U/ml penicillin and 100 μg/ml streptomycin at 37°C in a humidified environment containing 5% CO2. Exponentially growing cells were used for experiments.

### Cell viability and chemosensitivity assay

Cells (2000/well for AGS, 4000/well for N87) were seeded into 96-well plates and stained at the indicated time point using alamarBlue (Thermo scientific, FL, USA) according to the manufacturer's instructions. The optical density measured at 530/590 nm was used as an indicator of cell viability. For chemosensitivity assay, the medium was replaced by fresh medium with or without various concentrations of Cisplatin (CDDP, Calbiochem, CA, USA) (5, 10, 25, 50, 100 μM). Cell viability assay was performed after 48 hrs of treatment [61–63]. Six wells were counted for each drug concentration and at least three independent experiments were performed. The half maximal inhibitory concentration (IC50) value was defined as the concentration that resulted in a 50% reduction in cell growth compared with growth of the control.

### Plasmids construction, lentiviral and retroviral infection

The plasmid pLNCX/GLI1 (with a Myc tag) was previously constructed in our laboratory [51] and pLKO.1/shGLI1 and their corresponding control plasmid pLKO.1/shCtrl were from the Broad Institute. Expression plasmid pSIN4/ABCG2 was purchased from Addgene (#25983). The ABCG2/TM5-6 plasmid was kindly provided by Dr. Jian-ting Zhang at Indiana University School of Medicine, and subcloned into pBabe using *Eco*RI and *Bam*HI to obtain pBabe/TM5-6 (with Myc tag) [53].

To generate GLI1 knockdown, ABCG2 and TM5-6 overexpression cells, AGS and N87 cells were infected with lentivirus containing pLKO.1/shGLI1, pSIN4/ABCG2 and pBabe/TM5-6, respectively. Lentiviral packaging plasmids PRRE, RSV/REV and CMVG were co-transfected into HEK293T cells using Lipofectamine 3000 (Invitrogen, Carlsbad, CA, USA) for virus production and infection as previously described [64]. Twenty-four hours after infection, cells were selected with 1 μg/ml puromycin (Sigma, St Louis, MO, USA) or 1 mg/ml G418 (Invitrogen).

### Reverse transcriptase and real-time PCR

Total RNA was isolated from cells using TRIzol reagent (Sigma) according to the manufacturer's instructions. One microgram of total RNA was reverse transcribed into cDNAs using the First-Strand Synthesis Kit (Roche, Indianapolis, IN, USA). The abundance of transcripts in the cDNA samples was measured by real-time PCR with specific probes as instructed from the provider and previously described [65, 66]. All probes for real-time PCR were purchased from the Applied Biosystems.

### Western blot analysis

Cells were lysed in cell lysis buffer (50 mM Hepes, pH 7.4, 2 mM EDTA, 100 mM NaCl, 1% Glycerol, 1% Triton X-100) containing protease and phosphatase inhibitors. After separation by SDS-polyacrylamide gel electrophoresis and protein transfer onto PVDF membrane. Antibodies were used to incubate with the membrane [Rabbit anti-GLI1 antibody diluted at 1:1000 (from the Cell Signaling Technology, MA, USA); mouse anti-Myc 9B11 antibody (diluted at 1:1000, the Cell Signaling Technology); mouse anti-β actin antibody (diluted at 1:10000, Santa Cruz Biotechnology, Santa Cruz, CA, USA) and goat anti-rabbit-HRP/goat anti-mouse-HRP (diluted at 1:10000, Thermo scientific)]. Detection was performed with ECL Western blotting detection kit (Thermo scientific) as described previously [64, 66].

### Flow cytometry analyses

Single cells were dissociated using Accutase® (Gibco, CA, USA) and re-suspended in PBS containing 10% FBS. The cells were incubated with fluorescence-conjugated antibody against human CD24, CD33, CD44, CD90, CD133 and ABCG2 (all from Biolegend, CA, USA) for 30min at 4°C. After washing, cells were analyzed on FACSCalibur or FACSCanto II (Beckton Dickinson, Franklin Lakes, NJ, USA) [66].

For side population assay, single cells were re-suspended at 1 × 10^6^/ml in DMEM with 2% FBS and 10 mM HEPES, stained with Hoechst 33342 dye (final concentration 5 μg/ml, Invitrogen) and incubated at 37°C for 90 min with shaking. The cells were washed with ice-cold HBSS with 2% FBS and 10 mM HEPES, spined down at 4°C and re-suspended in ice-cold HBSS containing 2 μg/ml propidium iodide (PI, Invitrogen). An aliquot of cells was used as a negative control by adding ABC transporter inhibitor fumitremorgin C (FTC, 10 μM, Calbiochem) and incubated at 37°C for 30 min before addition of Hoechst 33342.

### Sphere formation assay

Cells were re-suspended in sphere formation medium (Neural Basal medium with 1× B-27, 20 ng/ml of EGF, 10 ng/ml of βFGF and 5 μg/ml of heparin), and plated on ultralow attachment 24-well plates with 2000 cells each well (Corning, Lowell, MA, USA) as described previously [67]. Sphere formation medium was changed every other day. After 7 days, spheres were counted and taken photos, or cells were dissociated with Accutase® and used for other experiments. The sphere forming efficiency was calculated by counting the number of spheres formed from 2000 cells.

### Chromatin immunoprecipitation (ChIP) assay

ChIP was performed as previously described [51]. In brief, cells at 80% confluence were cross-linked with formaldehyde (Sigma) at room temperature for 15 min. Formaldehyde was neutralized by addition of 125 mM glycine for 5 min. The cells were collected by centrifugation and rinsed in cold phosphate-buffered saline. The cell pellets were collected by centrifugation and then re-suspended in sonication buffer (1% SDS, 10 mM EDTA, 50 mM Tris-HCl, pH 8.1, 0.5 mM PMSF, and 100 ng of leupeptin and aprotinin/ml) and incubated on ice for 20 min. The samples were sonicated on ice with an Ultrasonics sonicator at setting 10 for 20-second pulses to yield an average length of 500-1000 bp for genomic DNA. The chromatin solution was pre-cleared with the addition of protein A/G plus agarose beads (from the Santa Cruz Biotechnology Inc.) for 30 min at 4°C. Prior to use, the protein A/G plus agarose beads were blocked with sheared herring sperm DNA (1 μg/μl) and bovine serum albumin (1 μg/μl) for at least 4 hr at 4°C. 10% of the supernatant was saved as total input, and the rest was immunoprecipitated with 5 μg of normal anti-mouse IgG (Biolegend), 5 μg of Myc-tag mouse monoclonal antibody (Cell signaling technology) or 5 μg of anti-Histone H3 polyclonal antibody (Biolegend) at 4°C overnight, and then incubated with protein A/G plus agarose beads for 1 hr. Immunoprecipitates were washed twice in TE buffer (10 mM Tris-Cl, pH 8.0, 1 mM EDTA, 1 mM PMSF), once with Low Salt Wash buffer (0.1% SDS, 1% Triton X-100, 2 mM EDTA, 20 mM Tris-Cl, pH 8.0, 150 mM NaCl, 1 mM PMSF), twice with High Salt Wash buffer (0.1% SDS, 1% Triton X-100, 2 mM EDTA, 20 mM Tris-Cl, pH 8.0, 500 mM NaCl, 1 mM PMSF), once with LiCl Wash buffer (0.25 M LiCl, 1% NP-40, 1% Sodium deoxycholate, 1 mM EDTA, 10 mM Tris-Cl, pH 8.0, 1 mM PMSF) and three times in TE buffer. Immunoprecipitates were eluted in Elution buffer (50 mM Tris-Cl, pH 8.0, 10 mM EDTA, 1% SDS, 200 mM NaCl) and incubated at 65°C for 30 min. DNA was purified by extraction with phenol/chloroform and precipitated with 1/10 volume of 3 M NaOAc (pH 5.3) and 2.5 volumes of ethanol. The DNA fragment was amplified by PCR using primers flanking the promoter region 1000 bp upstream. Primers used to PCR-amplify the ABCG2 gene chromatin were 5’-ATCCCATTCACCAGAAACCA-3’ and 5’–CGAACGGAATGAACCAGAGT-3’ resulting in a product size of 205 bp. Primers used to PCR-amplify 18s as an internal control were 5’-CAGCCACCCGAGATTGAGC-3’ and 5’-TAGTAGCGACGGGCGGTGTG-3’ resulting in a product size of 252 bp.

### Tumor xenografts and anticancer chemotherapy *in vivo*

All animal procedures were performed in accordance with a protocol approved by the Institutional Animal Care and Use Committee in Indiana University (Indianapolis, IN). To generate subcutaneous tumors, 5 × 10^6^ cells were re-suspended in 100 μl PBS and subcutaneously injected into NOD scid gamma (NSG) mice. Tumor volume (volume = 0.5 × length × width^2^) was measured with a caliper twice a week. When tumors reached 80-100 mm^3^ (as day 0 in this study), mice were injected intraperitoneally (i.p.) once a week with CDDP (4 mg/kg) or vehicle (PBS) control group for two weeks. On day 21, mice were sacrificed, and tumor xenografts were removed, weighted and fixed in 10% buffered formalin for further analysis.

### Immunohistochemistry (IHC)

Two ninety-dot tissue arrays were purchased from Shanghai Outdo Biotech Company (Shanghai, China). Formalin-fixed, paraffin-embedded sections were deparaffinized by xylene, rehydrated in ethanol and boiled in 10 mM citrate buffer (pH 6.0) for 30 min for antigen retrieval. Endogenous peroxidase was blocked by 3% H_2_O_2_ for 10 min for immunoperoxidase labeling. Sections were then incubated at 4°C overnight with primary antibodies against rabbit anti-ABCG2 (1:100, Abcam, Cambridge, UK). Incubation with corresponding secondary antibody (ImmPRESS universal peroxidase reagent, Vector Lab, CA, USA) and the peroxidase-antoperoxidase complex was visualized by DAB kit (ImmPACT DAB Peroxidase Substrate, Vector Lab), as previously described [68]. Two individuals (B.Y. and J.X.), who had no prior knowledge of the clinical pathologic data of the patients, examined the stained sections independently. Positive membrane ABCG2 staining was assessed by the intensity of stained cells and determined in two categories (negative and positive). The staining intensity was classified from 0 to 3+ as follows: 0, no staining; 1+, < 25% of the section was stained; 2+, 26% - 50% of the section was stained; 3+, > 50% of the section was stained. Scores of 1+, 2+ and 3+ were considered to be positive.

### Statistical analysis

Data are presented as mean ± SD. from at least three independent experiments. IC50 values of CDDP were calculated with the GraphPad Prism software. Survival data were analyzed using the Kaplan-Meier method with a log-rank test for comparison of survival curves. Statistical comparisons between two groups were performed using a two-tail unpaired t-test or the chi-square test, with *P* values of < 0.05 indicating statistically significant difference.

## SUPPLEMENTARY MATERIALS FIGURES


