# Supplementary material for: GLI1-mediated regulation of side population is responsible for drug resistance in gastric cancer
Source: Oncotarget. 2017 Mar 14;8(16):27412–27. doi: 10.18632/oncotarget.16174 (PMC5432345; doi:10.18632/oncotarget.16174)
Supplement: Supplementary file 1 [file oncotarget-08-27412-s001.pdf]

GLI1-mediated regulation of side population is responsible for drug resistance in gastric cancer

SUPPLEMENTARY MATERIALS

SUPPLEMENTARY FIGURES

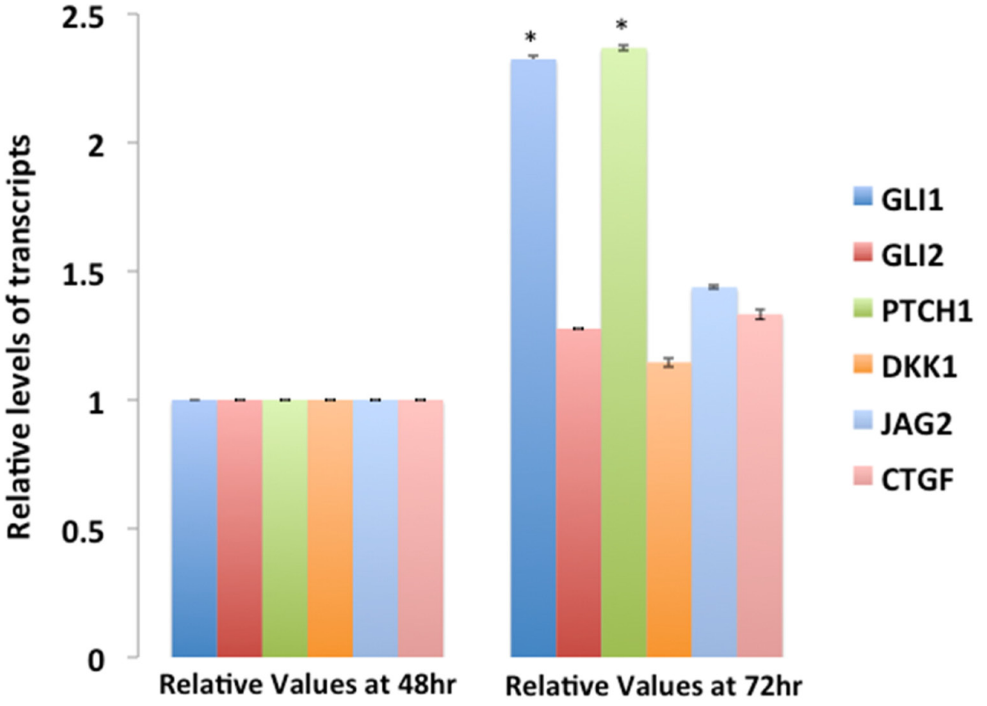

Supplementary Figure 1: Comparison of gene expression between 48hr and 72 hr treatment of CDDP in N87 cells. Cells were treated with the IC50 dose of CDDP for 48 and 72 hrs, and the gene expression was detected by real-time PCR. In comparison of the two time points, only Gli1 and PTCH1 show the significant changes. This result confirms that Hh signaling is activated after CDDP treatment.

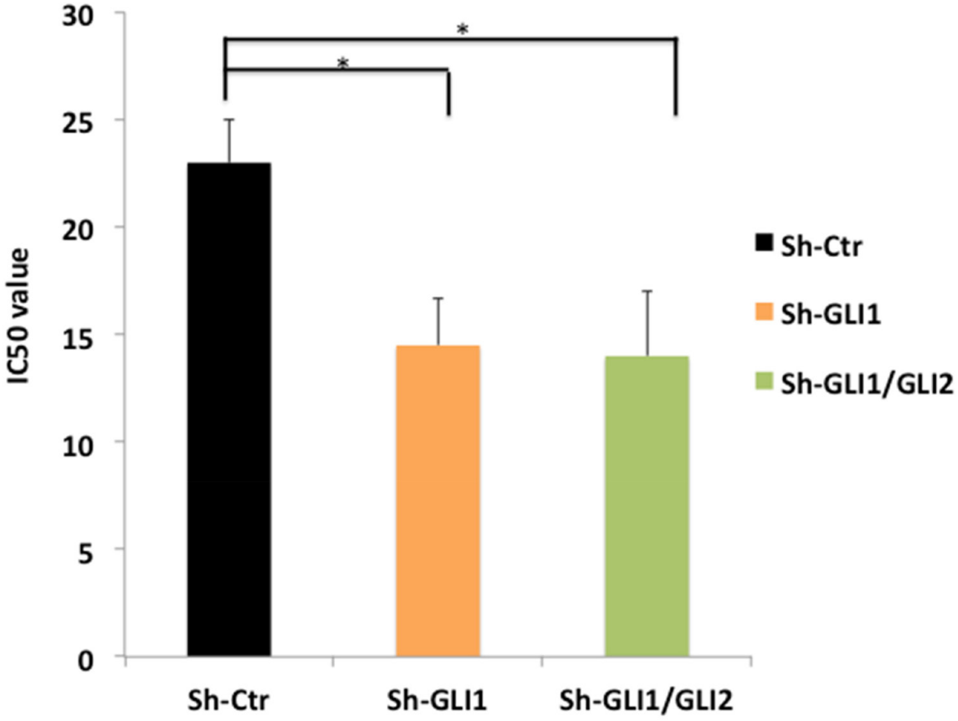

Supplementary Figure 2: Comparison of the IC50 between Sh-GLI1 and Sh-GLI1/GLI2 expressing N87 cells. Different types of shRNAs (Sh-CTR, Sh-GLI1 and Sh-GLI1/GLI2) were expressed in N87 cells, and the stable lines were treated with CDDP to measure the IC50. While both Sh-GLI1 and Sh-GLI1/GLI2 expression significantly reduced the IC50, there were no differences between Sh-GLI1 and Sh-GLI1/GLI2 expressing cells. This result indicates that the forward positive feedback loop of GLI1 is the major downstream mediator for reduced CDDP sensitivity.

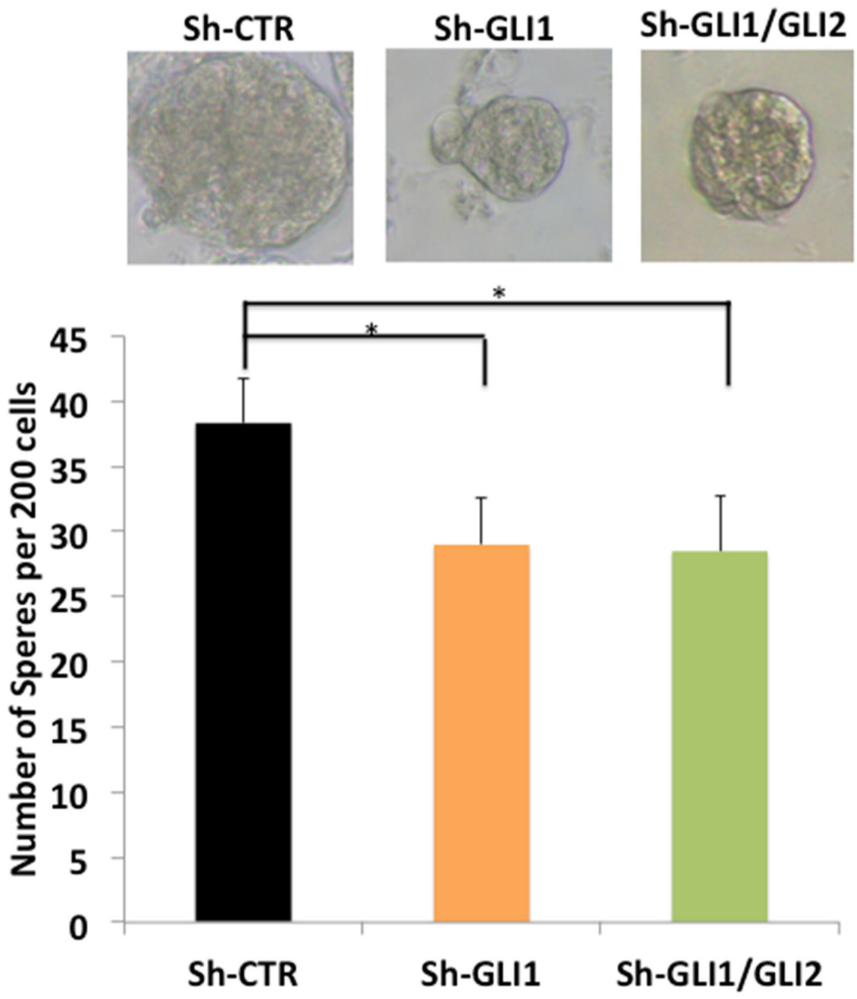

Supplementary Figure 3: Tumor sphere formation in N87 cells following expression of different ShRNAs. The up panel shows the typical images of tumor spheres whereas the low panel shows the number of tumor spheres. N87 Cells expressing Sh-GLI1 and Sh-GLI1/GLI2 all had significantly reduced tumor sphere number than the Sh-CTR expressing cells, but there were no differences between Sh-GLI1 and Sh-GLI1/GLI2 expressing cells. This result is consistent with Supplementary Figure 2 that the forward positive feedback loop of GLI1, the focus for the rest of the manuscript, is the major downstream mediator for reduced CDDP sensitivity.

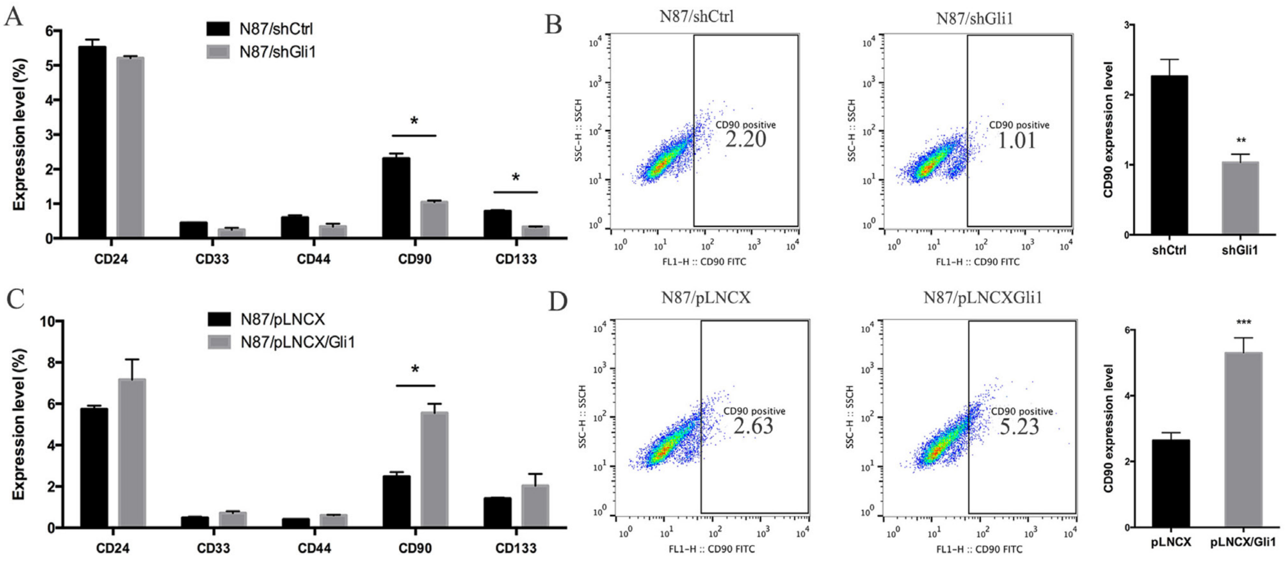

Supplementary Figure 4: Cell surface marker expression. (A) Comparison of CD24, CD33, CD44, CD90 and CD133 cell surface markers between N87/shCtrl and N87/shGLI1 cells. **(B)** The distribution of CD90 positive cells in N87/shCtrl and N87/shGLI1 cells by flow cytometry. **(C)** CD24, CD33, CD44, CD90 and CD133 expression levels in N87/pLNCX and N87/pLNCX/GLI1 cells. **(D)** CD90 expression level in N87/pLNCX and N87/pLNCX/GLI1 cells.

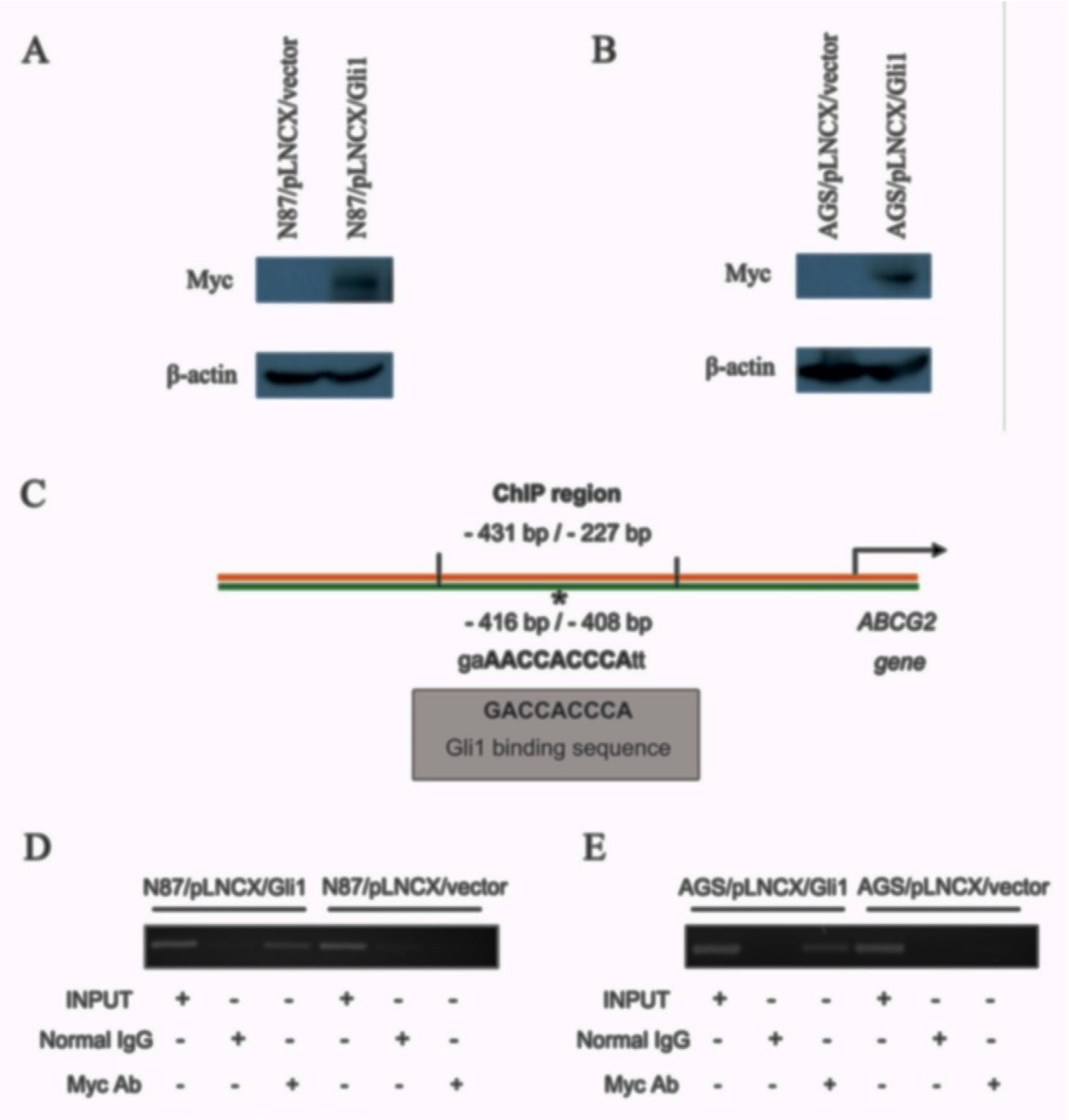

Supplementary Figure 5: Gli1 directly binds to the promoter of ABCG2. **(A)** Detection of ectopic GLI1 expression by the tag (Myc) antibodies in Western blot analysis in N87/pLNCX/GLI1 cells. N87/pLNCX/vector was the control cell line. **(B)** Western blot analysis of ectopic GLI1 expression in AGS/pLNCX/GLI1 cells, with AGS/pLNCX/vector as the control. **(C)** A schematic diagram of the 9 base pairs sequence of the potential GLI1 binding site in the ABCG2 promoter. **(D)** ChIP analysis in N87/pLNCX/vector and N87/pLNCX/GLI1 cells using Myc-tag antibody, normal IgG as an internal control. **(E)** ChIP analysis in AGS/pLNCX/vector and AGS/pLNCX/GLI1 cells.

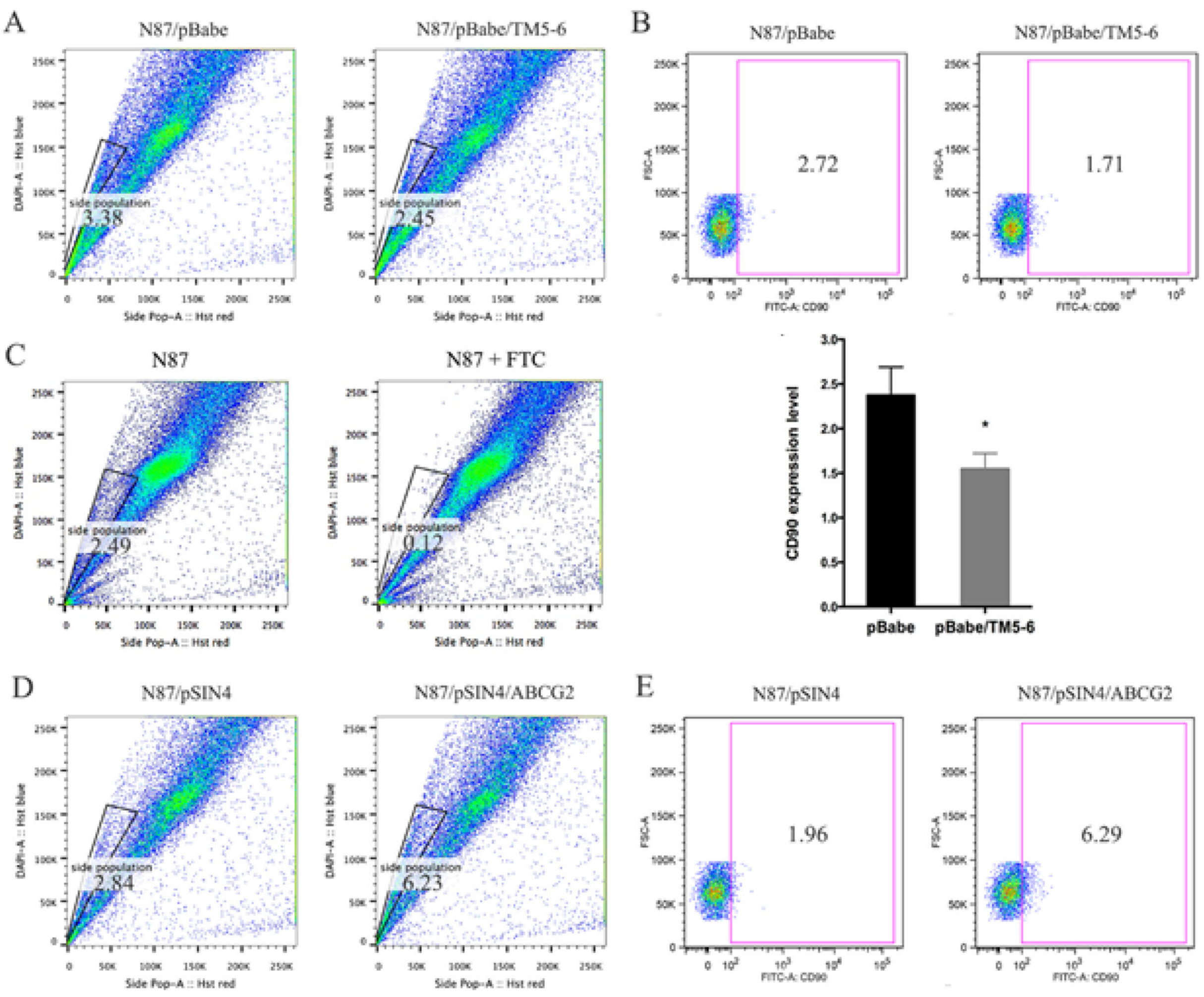

Supplementary Figure 6: Flow cytometry analyses. **(A)** Representative images of side population in N87/pBabe and N87/pBabe/TM5-6 cells. **(B)** CD90 distribution in N87/pBabe and N87/pBabe/TM5-6 cells. **(C)** Side population analysis in N87 cells with or without FTC (10 μM). **(D)** Representative images of side population in N87/pSIN4 and N87/pSIN4/ABCG2 cells **(E)** CD90 distribution in N87/pSIN4 and N87/pSIN4/ABCG2 cells.

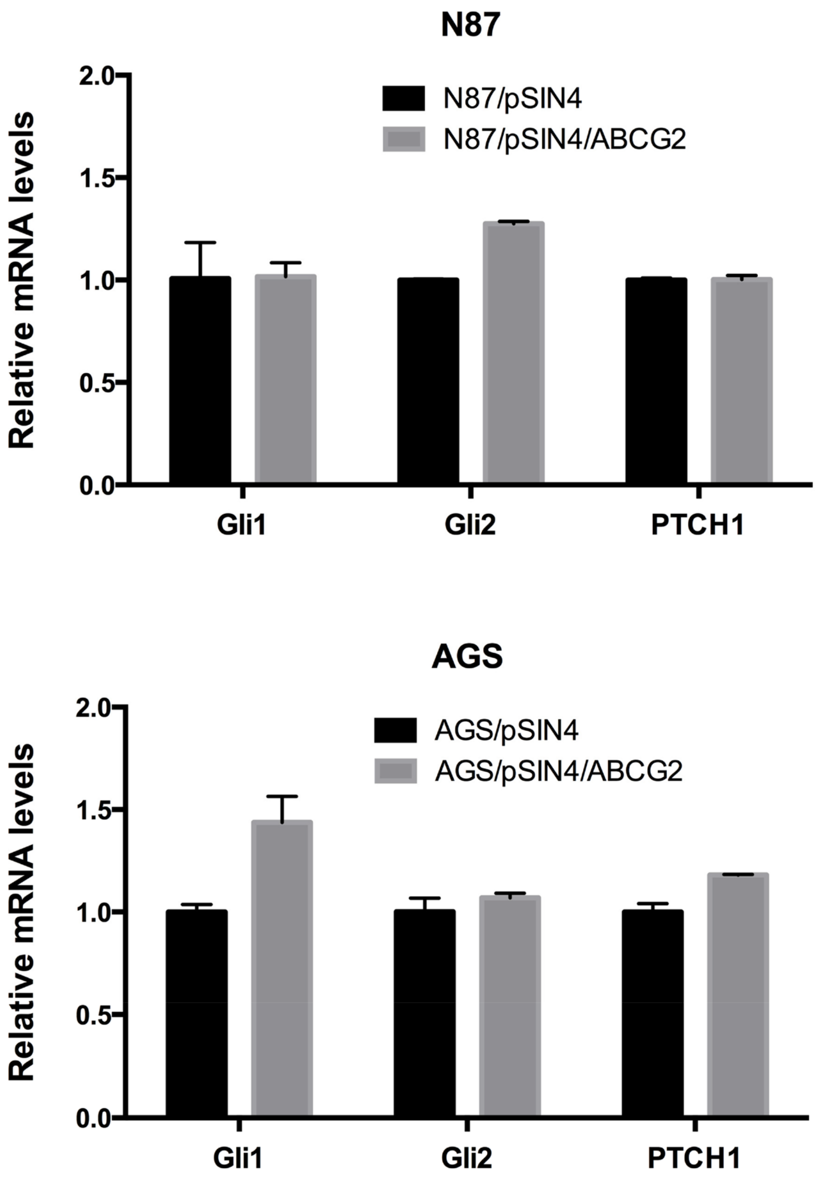

Supplementary Figure 7: ABCG2 does not regulate Hh signaling activity. Real-time PCR analysis of *GLI1*, *GLI2* and *PTCH1* in N87/pSIN4 and N87/pSIN4/ABCG2 cells (upper), AGS/pSIN4 and AGS/pSIN4/ABCG2 cells (lower) did not reveal significant changes in these Hh signaling target genes.

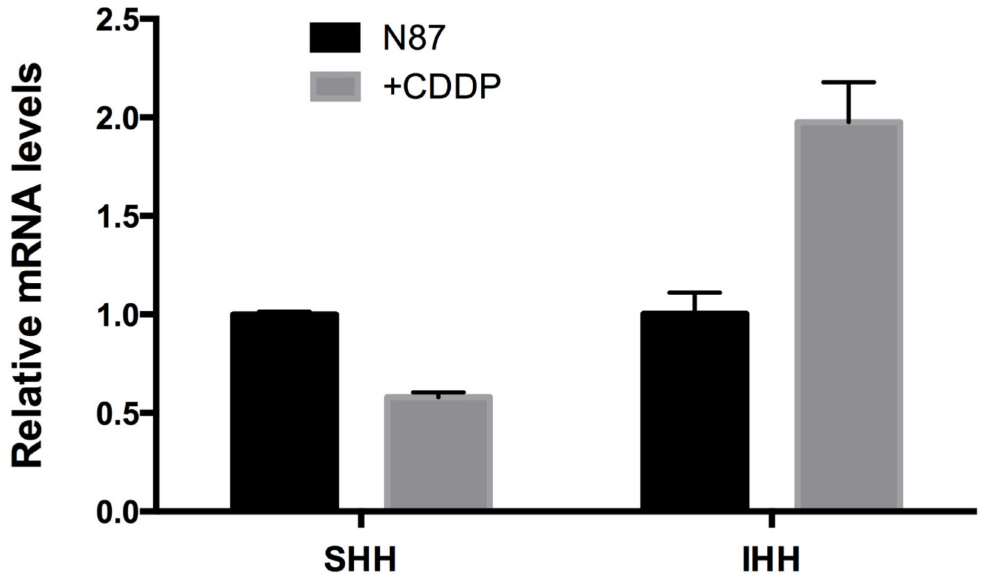

Supplementary Figure 8: Real-time PCR analysis of SHH and IHH expression. N87 cells with 25 µM CDDP treatment had no significant changes in SHH and IHH expression, suggesting that elevated Hh signaling may not be resulted from SHH or IHH overexpression.

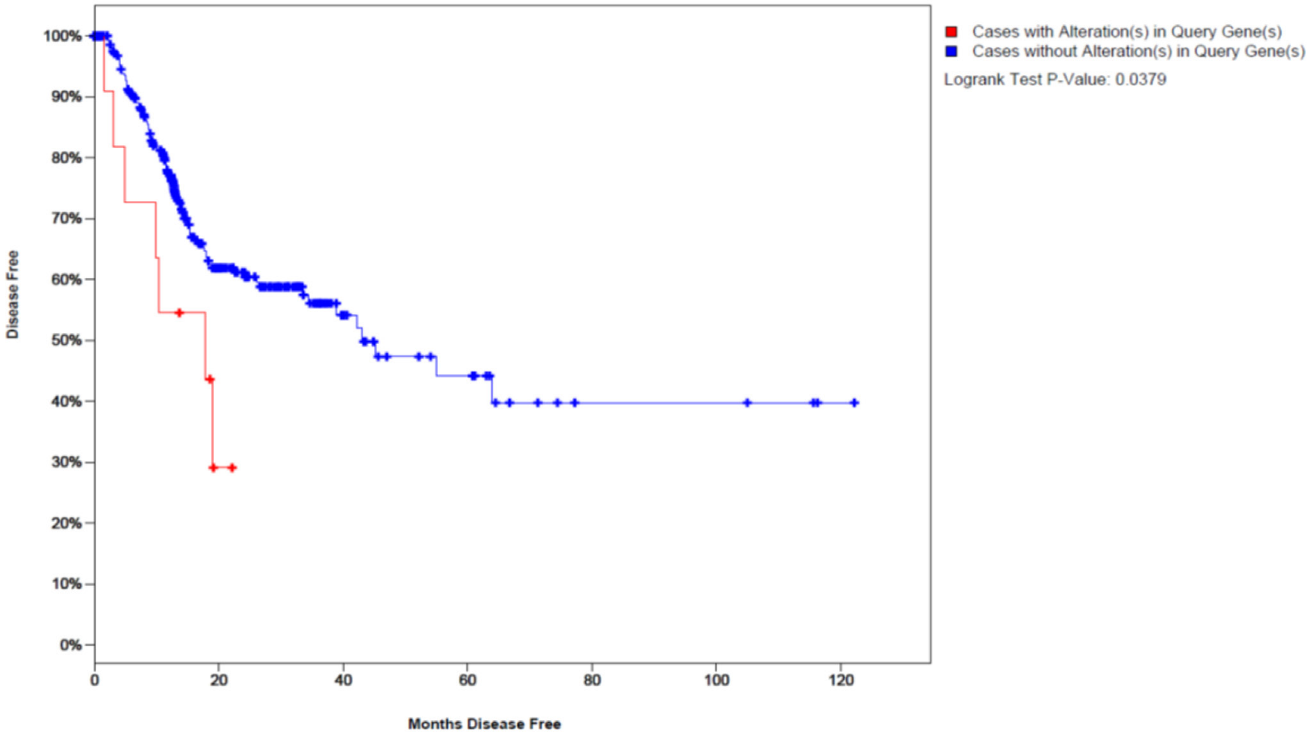

Supplementary Figure 9: Analysis of CTGA data on the association between GLI1 expression and gastric adenocarcinoma patient survival. A cohort of 415 patients with sequencing data from the original 487 specimens was analyzed using CBioportal Cancer Genomics. The data on cancer relapse were provided by the CTGA. Disease-free survival of the patients was correlated with high or low GLI1 expression, and the P value=0.0379.
